# What every dental practitioner should know about how to examine patients with dental implants

**DOI:** 10.1038/s41415-023-5574-6

**Published:** 2023-03-10

**Authors:** Fadi Barrak, Daniel Caga, StJohn Crean

**Affiliations:** 41415132186001 Specialist Oral Surgeon, University of Central Lancashire, UK; Course Lead MSc Clinical Implantology, University of Central Lancashire, UK; Founding CEO, VSS Academy, UK; 41415132186002 Associate Dentist, Birmingham, UK; 41415132186003 Pro Vice-Chancellor (Research and Enterprise), University of Central Lancashire, UK

## Abstract

Dental implants are a common treatment modality provided in both primary and secondary care settings. It is increasingly common for a general dental practitioner to see patients with implant-retained restorations. This article suggests an implant safety checklist for general dental practitioners to help them examine an implant-retained prosthesis.

## Key points

- Highlights the importance of examining dental implants.
- Informs how to examine a dental implant.
- Advises on what to do when we find problems with dental implants.

## Introduction

In contemporary dental practice, there are very few clinical settings in which a dental practitioner can avoid seeing patients who have had implant treatment and require long-term maintenance. In addition, dental implant therapy has been recognised as an increasingly high-risk area for civil negligence claims and General Dental Council fitness to practise hearings.^1^ Despite this, the training for implant dentistry remains an informal and optional postgraduate endeavour. In the authors' opinion, this highlights a potential mismatch between the knowledge and skill requirement of the general dental practitioner (GDP) in managing such patients and the training provided at undergraduate, foundation training (FT) and general postgraduate (PG) levels.

Addy *et al.* in 2008 investigated the provision of dental implant teaching in UK and Ireland undergraduate dental schools.^2^ An online questionnaire was developed and distributed to 15 dental schools, exploring the current and future trends in implant dentistry education at undergraduate level. The results indicated that two schools did not provide any undergraduate implant dentistry teaching, while 13 schools did provide teaching; most of which (eight schools) consisted of lecture-based or phantom head events. The authors concluded that, even though the teaching of dental implantology had increased relative to previous studies, all respondent schools anticipated a need to increase training within a five-year period, to better prepare graduates for clinical practice.^2^ A qualitative study by Ray *et al.* (2018) involving three UK dental schools investigated the contributing factors which influence dental graduate preparedness in final year dental students.^3^ This study utilised focus groups and semi-structured interviews and thematic analysis of the data was completed. The students reported that the most important factor affecting their preparation was clinical exposure. With the results of Ray *et al.* in mind, it may be suggested that, most newly qualified dentists would (anecdotally) agree that they feel ill-prepared for appropriately referring a patient for implant treatment, let alone examining the health of a patient's dental implant.^3^

Jayachandran *et al.* (2015) investigated the opinions of GDPs in a group of West Midland practices about their opinion on the current level of implant education at both undergraduate and postgraduate levels.^4^ This survey had a 95.6% response rate (87/91), where 77% of respondents reported they were only taught theoretical aspects of dental implantology at undergraduate level, the majority of which they reported as being inadequate.^4^

Chin *et al.* (2019) evaluated the understanding of peri-implant health among dental hygienists and therapists in Wales.^5^ Results revealed that although 92% (n = 85) of respondents reported implant care was in their remit of service, only 64% (n = 54) completed clinical assessments of peri-implant health.^5^ Interestingly, 83% (n = 76) felt postgraduate training in maintenance should be obligatory,^5^ while 62% and 11% of respondents reported being somewhat and not confident, respectively, in clinically assessing dental implants.^5^ Additionally, 20% were somewhat confident in instructing patients in methods of plaque control. With respect to supragingival debridement of the dental implant supported structures, 38% and 3% of respondents reported being somewhat confident and not confident.^5^ Finally, for the provision of subgingival debridement procedures, 45% and 18% of respondents reported being somewhat confident and not confident.^5^ Although not directly involving dental students or qualified dentists, this study did highlight the clinical exposure of dental care professionals within a primary care practice environment and furthermore demonstrated a reluctance and lack of confidence in the provision of peri-implant maintenance. Further research would help to evaluate whether such issues pervade the wider dental profession.

Within the UK, dental implants are being placed in a wide range of clinical settings, and as a result, GDPs will inevitably come across patients who have received implant therapy and should therefore be examining dental implants. This will demand a minimum standard for implant training at undergraduate, FT and PG level in order to be able to provide the required professional care. This is even more important when considering that, in a 2017 survey of consultants in restorative dentistry within the UK, only 64% reported they would accept a referral for peri-implant disease.^6^

On review of the dental literature, the terms success and survival rates have been a source of confusion for many years.^7^ According to the International Team of Implantology (ITI), the definition of survival indicates the implant is simply present at follow-up but its condition is not specified; while the definition of success indicates the presence of the implant at the follow-up appointment and complications are absent.^8^ Implant survival rates have been reported as 81.73-100% at three years, 74.09-100% at four years, 76.03-100% at five years and 69.63-98.72% at seven years.^9^

Several factors can therefore influence the long-term survival of dental implants.^10^

Both biological and technical complications can affect the clinical outcomes of dental implant therapy. Biological complications involve inflammatory conditions, such as peri-implant mucositis and peri-implantitis (see Box 1 for definitions), as well as soft tissues lesions, such as pain, swelling, hyperplasia and fistula formation.^11^

Technical complications affecting dental implants include: i) fracturing of the implant itself;^8^ ii) fracture of veneering material; iii) abutment or screw loosening; and iv) loss of retention.^11^

It is clear from the literature that dental implants can and do fail. Failure can be classified as early or late. Early implant failure occurs as a result of unsuccessful osseointegration, while late failure occurs after successful osseointegration.^12^

One of the major causes of late implant failure has been attributed to peri-implantitis (Box 1),^13^ which can progress from peri-implant mucositis if not controled.^14^ Therefore, in order to ensure long-term stability of a dental implant, it is vital to monitor and maintain their peri-implant health, as well as to identify and treat any associated disease as soon as possible.^9^

Dental implants need regular and ongoing monitoring and maintenance.^15^ Qualitative research suggests that GDPs may be unwilling to treat patients who had their dental treatment performed elsewhere.^16^

Dental professionals would benefit from having access to an easy-to-use checklist on how they should examine a dental implant and recognise potential problems in the primary dental care environment, thereby improving early diagnosis of peri-implant mucositis, peri-implantitis and the long-term prognosis of the implant.

Box 1 Definitions of peri-implant health, peri-mucositis and peri-implantitis

- **Peri-implant health**: the absence of peri-implant signs of soft tissue inflammation (redness, swelling, profuse BOP) and the absence of further additional bone loss following initial healing.
- **Peri-implant mucositis**: the presence of peri-implant signs of inflammation (redness, swelling, line or drop of bleeding within 30 seconds following probing) but no additional bone loss following initial healing.
- **Peri-implantitis**: the presence of peri-implant signs of inflammation, radiographic evidence of bone loss following initial healing, and increasing probing depth as compared to probing depth values collected after placement of the prosthetic reconstruction. However, in the absence of previous radiographs, radiographic bone loss of ≥3 mm in combination with BOP and probing depths ≥6 mm is indicative of peri-implantitis.

## Essential stability of oral health

Any patient referred for elective implant treatment must have all underlying active dental disease diagnosed and stabilised or treated before implant therapy.^17^ Stable oral health also includes a stable occlusion and good periodontal health. A short period of 3-6 months for reviewing and recording evidence of periodontal status may miss fluctuations in periodontal health (bleeding on probing [BOP] and pocket depths), reflecting the variability in the patient's control, motivation and physiological ability to maintain such high levels of periodontal health. Implant treatment in the presence of active periodontal disease is contraindicated due to the increased risk of peri-implantitis, hence the need to stabilise the patient's periodontal health before commencing implant treatment.^1^These patients must also be warned that they are still at a higher risk of peri-implant disease, even if they have been stabilised pre-operatively.^15^

## Guide to examining the dental implant patient

There are risk assessment tools for biological complications around dental implants by Heitz-Mayfield, also referenced in the ITI treatment guide (volume 13) *Prevention and management of peri-implant diseases.*^18^ Here, the authors provide a suggested ten-point checklist for the GDP to use when recording the clinical notes, with a view to help with an accurate history of implant health status and identification of any events which would demand interventional steps.

## Implant examination checklist for GDPs

The following is a mnemonic to help with remembering the ten points of the implant examination checklist: Safety Is Overseen By Dentists On Monitoring Clues From Reviews.

### Satisfaction

Record whether the patient is happy with the prosthesis or if they have any symptoms or complaints. Factors affecting patient satisfaction may include the prosthesis itself (overall shape and shade, clean ability), as well as the soft tissue aesthetics (presence of black triangles, any metal show-through in the gingival tissues). A 2017 systematic review investigated the differences in aesthetic satisfaction between clinicians and patients when evaluating single tooth implant-supported restorations.^19^ Here, 11 articles, from an original 555, were included in the study. The results indicated that patients were 43-93% and 81-96% satisfied with the peri-implant soft tissue and implant restorations, respectively, while clinicians were more critical of the aesthetic outcomes.^19^ Critically, however, no meta-analysis was completed due to limitations in the studies included in the review, the interventions and aesthetic assessment methods used within them.

### Inflammation in surrounding tissues

This refers to any sign of inflammation and tenderness of the adjacent alveolar region surrounding the implant site. It may be an indication of inflammation within the coronal aspect of the gingival tissues or along the length of the fixture within the alveolus (indicative of peri-implant mucositis or peri-implantitis). Such findings would require further investigation with a periapical radiograph, detailed pocket charting and a referral to the clinician who placed the implant or someone with further training in the management of dental implants.

### Oral hygiene

Poor oral hygiene is a good indicator for future peri-implant disease. Plaque accumulation onto implant surfaces results in peri-implant mucositis.^20^ Retrospective evidence indicates that, if untreated, peri-implant mucositis can convert into peri-implantitis.^14^ When considering patient-performed plaque control, a systematic review highlighted three main methods, which may include mechanical removal (electric or manual brushing), chemical disruption using adjunctive anti-microbials, and triclosan-containing toothpastes.^21^ However, there is a lack of evidence for an accepted standard of care; the authors therefore suggest that tailored oral hygiene regimes should be implemented for each patient, considering both mechanical and chemical plaque disruption. Such regimes would have to consider the number of implant fixtures and the types and designs of prostheses being placed, as this will ultimately influence the type of patient-performed cleaning that will be required. For instance, a single implant crown will require the use of toothbrushes and interdental brushes, while an implant-retained bridge may require the use of super floss as well.

### Bleeding on probing

The *Consensus report of the sixth European workshop of periodontology* highlighted that it is essential to probe dental implants.^22^ The GDP can be reassured that conventional probing with light force (0.25 N) does not harm the peri-implant tissues^22^ and is recommended at least once a year.^13^ Both plastic and metal probes can be used. The authors suggest that GDPs utilise a probe they are familiar with to standardise their probing technique.

**Fig. 1:**
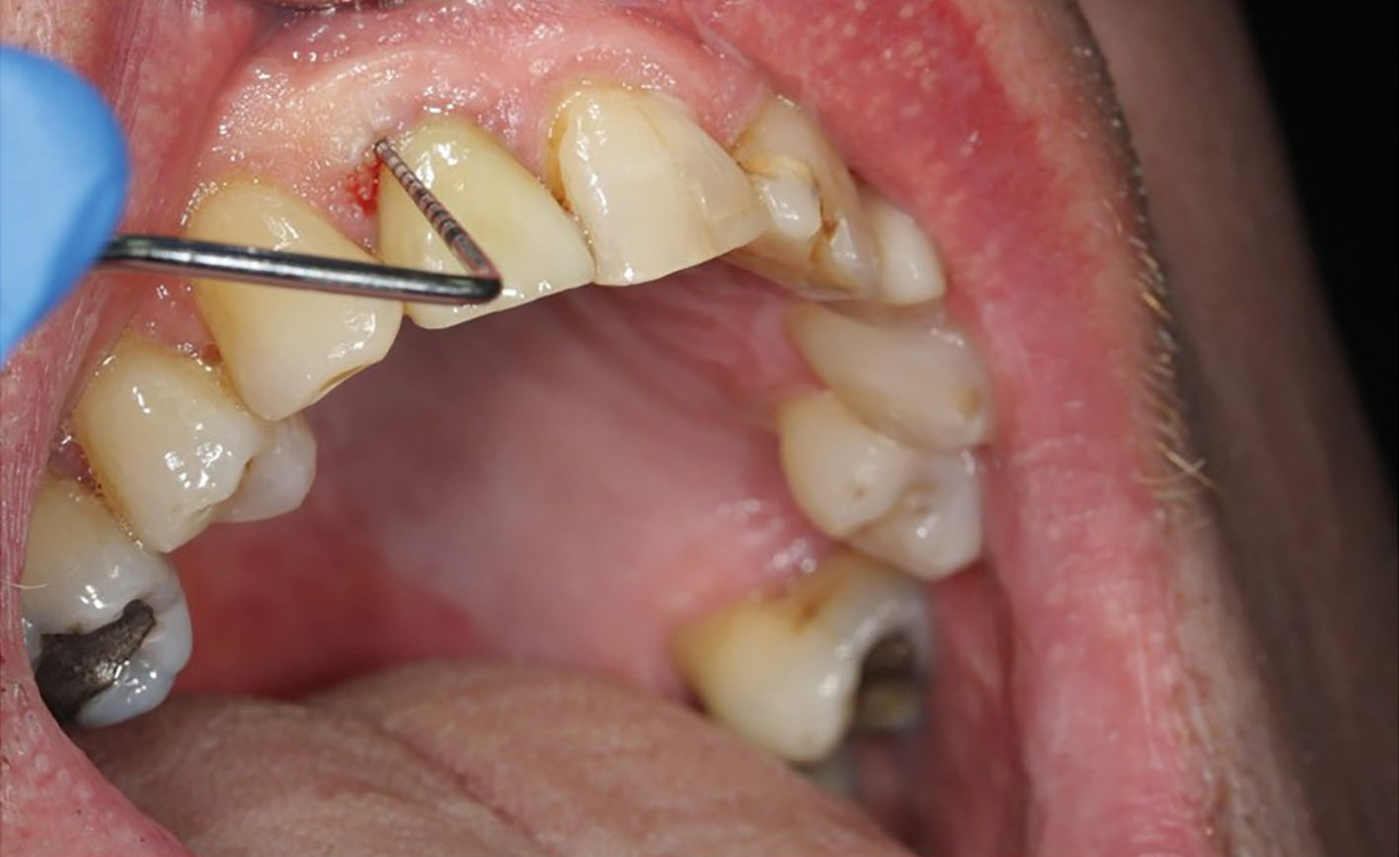
Image depicting evidence of BOP after gentle probing around the upper right central incisor implant crown. Note the 4 mm depth of the pocket and the correct angulation of the probe. Image courtesy of Dr Sonu Thomas

Implants can be probed using light force at six-points, or a sweeping motion along the entire circumference^17^(Fig. 1).

BOP is a key early indicator of disease and is associated with several risk factors, including poor oral hygiene, cigarette smoking, a history of periodontal disease, excess cement and prosthetic design.^23^ However, it is difficult to distinguish between BOP caused by peri-implant inflammation and induced by trauma from probing. It is important for the GDP to recognise and record such findings in the clinical notes and to educate their patients on behavioural changes. Where necessary, changes in the prosthetic design may also be required. A clinical review of implant fixture is required within 2-3 weeks following intervention to assess the effectiveness of the measures put in place. If resolution of the BOP is not achieved following the first review, referral to the clinician who placed the implant or someone with further training in the management of dental implants should be considered.

### Deep pockets

Pocket depths around healthy implants should generally be <5 mm in depth.^13^ Recording the probing depth at the time of fitting the restoration is vital for providing a baseline record which can be used as a reference point for future comparisons and diagnosis of peri-implant disease^17^ (Fig. 1).

These should be available from the clinician who placed and restored the implant. In the absence of baseline records (periapical radiograph, pocket depths and bone levels), peri-implantitis may be diagnosed when radiographic evidence of bone loss ≥3 mm from the implant neck and probing depths ≥6 mm in conjunction with bleeding and/or suppuration is recorded.^13^ This finding would require referral to the clinician who placed the implant or someone with further training in the management of dental implants.

### Occlusion

As implants lack a periodontal ligament, they also lack the 'shock absorbing' ability of natural teeth.^24^ Recording an occlusal examination is vital in the assessment of dental implant restorations. This should be completed at the restoration appointment, as well as at future review appointments, as naturally, a patient's occlusal scheme may change (that is, in the case of tooth surface loss or where dental extractions occur). However, the literature surrounding dental occlusion and dental implants is limited and an accepted standardised occlusal scheme is lacking. An occlusal assessment should include both static and dynamic functions. The patient's static occlusion would consider the function of the implant prosthesis during maximum intercuspation, while dynamic functions include anterior protrusive and lateral excursive movements. The occlusal prescription is dependent on the type of implant prosthesis placed. For example, with a single implant in a dentate patient, occlusal contacts in excursive movements may be avoided, whereas in a full arch restoration, this would not be possible. Clinical photographs of the occlusal contacts can be invaluable as a record in the patient notes for monitoring occlusal changes at subsequent appointments.

**Fig. 2:**
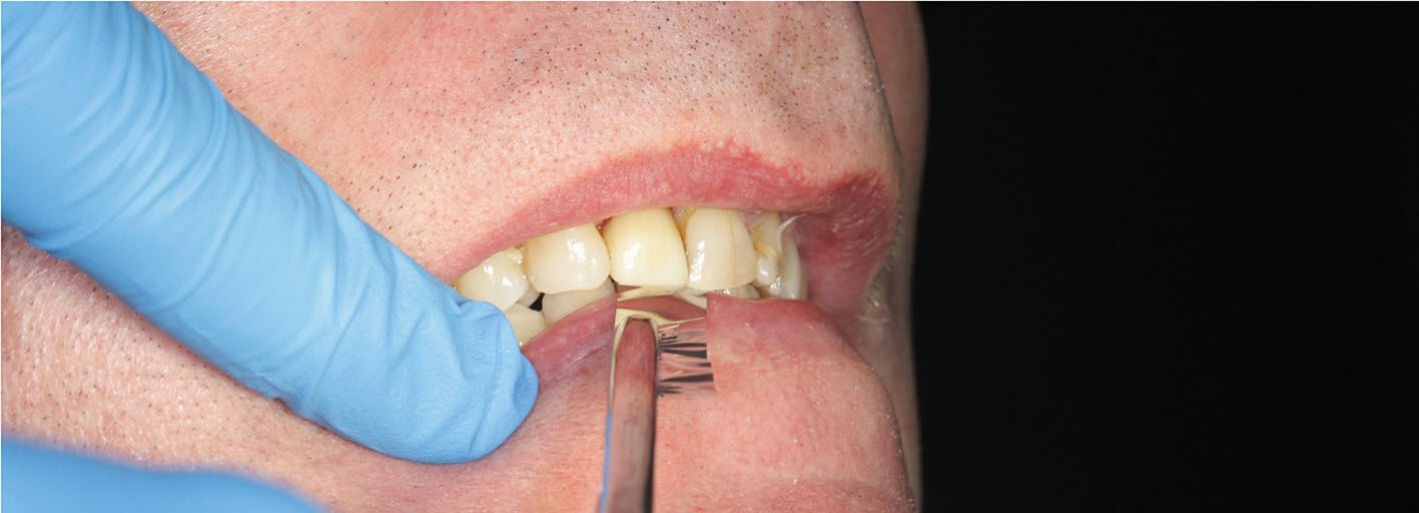
Image showing Shimstock foil being pulled through between the upper right central incisor implant crown and the opposing natural tooth in maximal inter-cuspal position. Image courtesy of Dr Sonu Thomas

During an occlusal assessment of a dental implant restored with a crown opposing a natural tooth, Shimstock foil can be used, with the patient firstly biting down lightly, during which the foil should pass through between the implant restoration and the opposing natural tooth. This is then repeated with the patient fully closing and clenching, during which the Shimstock foil contact should be held (Fig. 2). The occlusal assessment should also include any signs of occlusal overload on the implant prosthesis.

Furthermore, a review of the natural dentition is also required, highlighting any signs of occlusal wear and mobility.

If any change in the occlusion is noted then either a chairside adjustment can be made,^17^ or a referral made to the clinician who placed or restored the implant or someone with further training in the management of dental implants.

In the presence of occlusal overload, it may be necessary to provide an occlusal appliance for the patient. However, the evidence for the type of occlusal splint provided in implant dentistry is limited and may be based more on the clinician's opinion.^25^ It is the authors' opinion that, should an occlusal appliance be provided, a full coverage, hard occlusal guard providing a mutually protective occlusal scheme would provide better, appropriate and controlled occlusal protection.

### Mobility

**Fig. 3:**
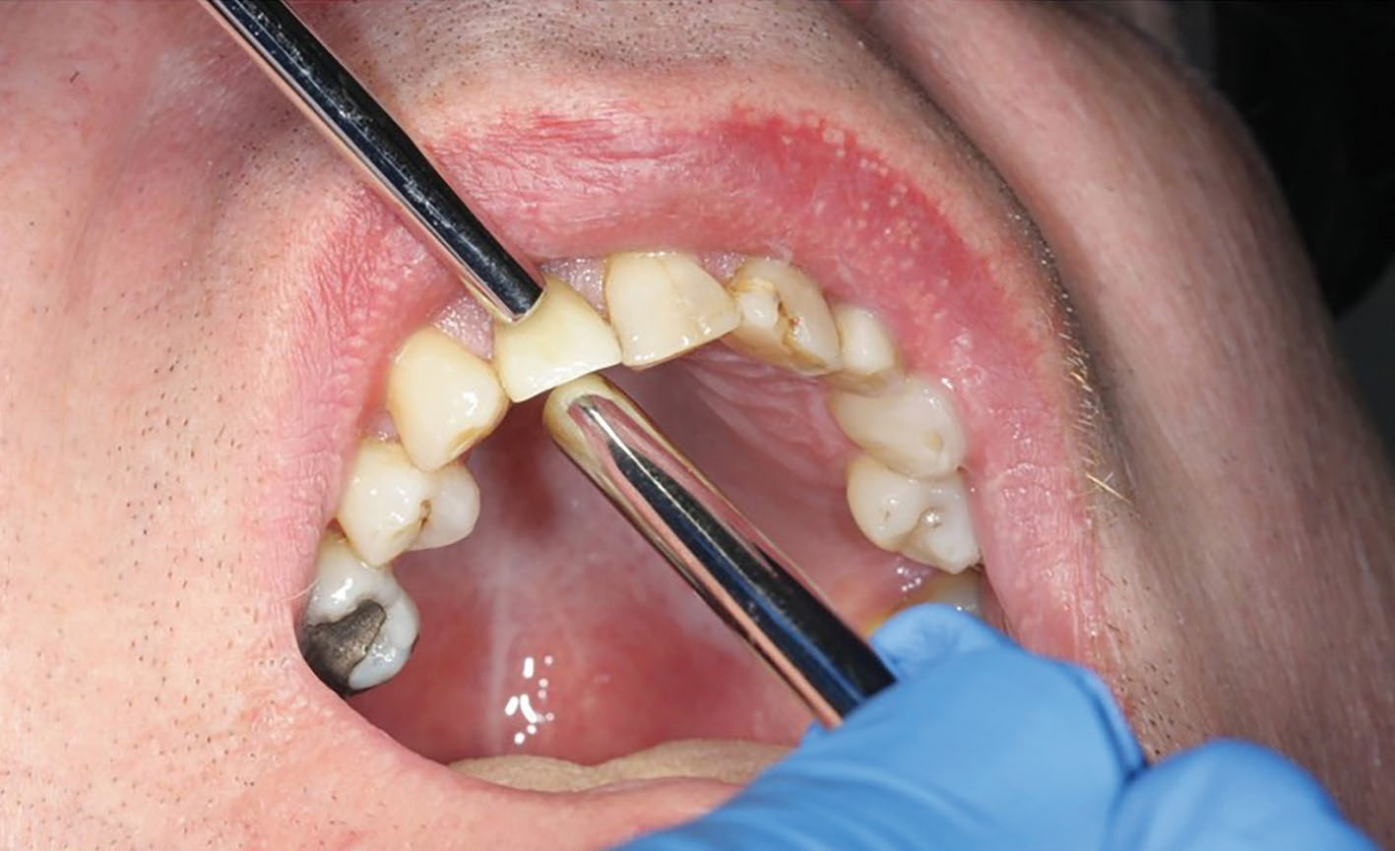
Image showing technique for examining the mobility of the upper right central incisor crown using the dental mirror and probe handles for more accurate feedback compared to using direct manual assessment. Image courtesy of Dr Sonu Thomas

Mobility may involve the dental implant fixture itself or the components used to restore it (that is, abutment screw, crown or bridge components). Any mobility should be investigated further and dealt with quickly, as this can rapidly deteriorate, resulting in inflammation, subsequent crestal bone loss and peri-implantitis. Mobility may also lead to fractures of the restorative component (such as the abutment screw). Mobility is best assessed using gentle pressure with an instrument (that is, dental mirror handle) on the implant crown as opposed to direct finger pressure, which can mask or give a false impression of movement (Fig. 3).

Pjeturrson *et al.* (2012) reported that, over a five-year period, the rates of abutment or screw loosening, and loss of retention of cemented implant-supported fixed dental prostheses, were 5.3% and 4.7%, respectively.^26^ If mobility of the implant crown is confirmed, then this should be investigated by a suitably trained clinician and therefore a referral may be required. Mobility of the actual implant fixture would usually cause some discomfort from the tissues surrounding the implant, while a loose abutment or crown may not impinge on soft or hard tissues and therefore be asymptomatic.

### Contacts points

The lack of tight contact points can result in food impaction and subsequent caries in adjacent natural teeth, as well as gingival inflammation, peri-implant mucositis and peri-implantitis. Even if the contact points were perfect at the time of the fit of the prostheses, teeth anterior to the implant can drift mesially, thereby opening a gap for food impaction. Hence, checking for the presence of tight contact points at each implant review is important. The integrity of the contact point can be recorded using clinical photographs and also by using floss or fine (12 μm) articulating paper between the contacts and recording this.

A discussion can be held with the patient regarding either altering the implant prosthesis or neighbouring natural teeth.^27^

### Framework integrity and emergence profile

GDPs should be reviewing the integrity of the implant prosthesis. Pjeturrson *et al.* reported that the most frequent complication over the five-year observation period was fracturing of the veneering material at a incidence of 13.5%. With respect to screw-retained prostheses, loss of the access hole restoration was reported as 5.4%.^26^

**Fig. 4:**
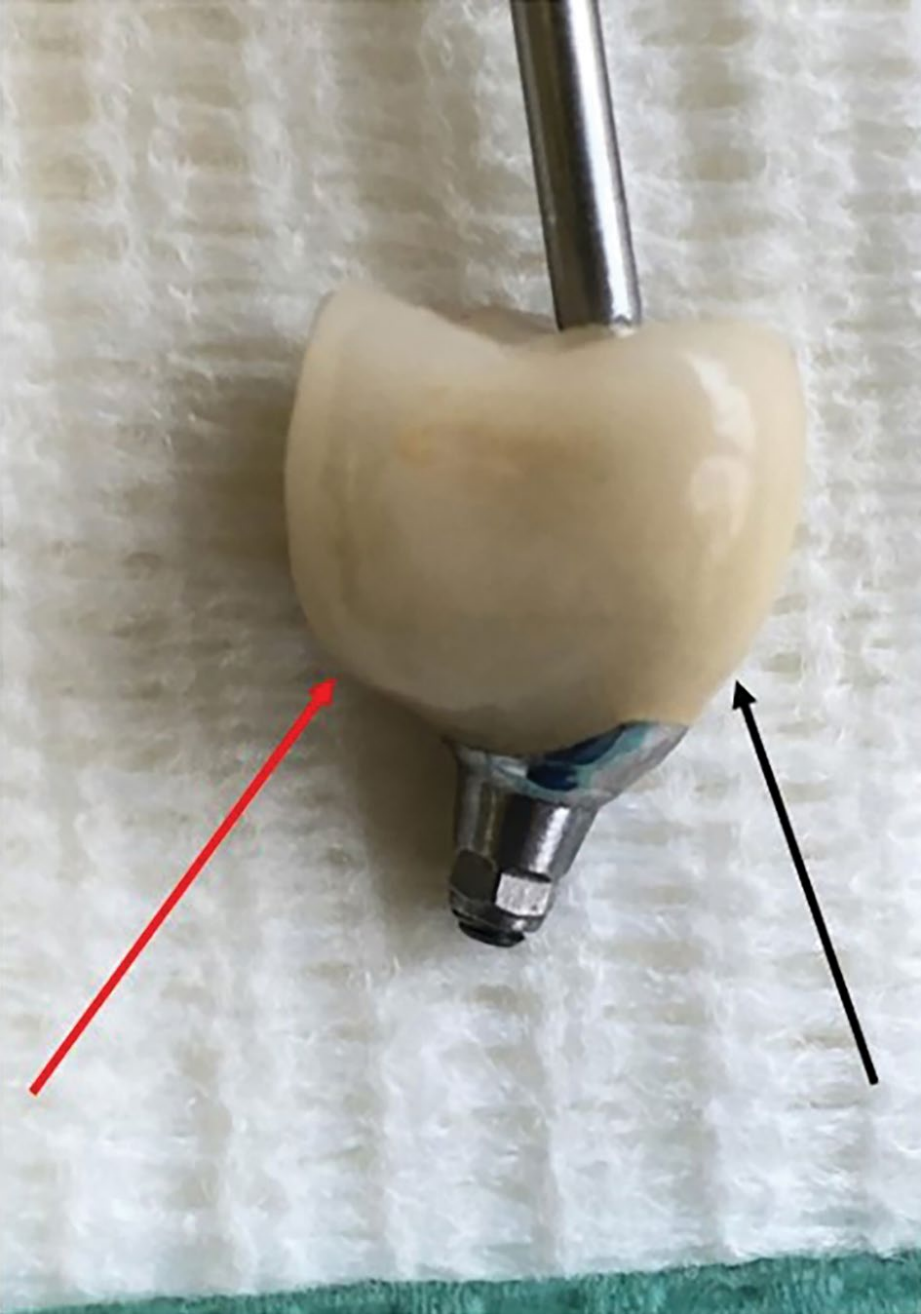
Image illustrating how a poorly contoured emergence profile (red arrow) on an implant crown makes it difficult for both the patient to clean sufficiently and the clinician to adequately probe the fixture to assess the integrity of the tissues, BOP and pocket depth, compared to a smooth emergence profile (black arrow)

The GDP should also consider the emergence profile of the implant restoration (Fig. 4). The peri-implant soft tissue architecture is different to that of a natural tooth, as a lack of Sharpey's fibre attachments to the implant surface results in the peri-implant soft tissues being less resistant to clinical probing and biofilm penetration compared to the natural dentition. Proper restorative emergence profile design is essential to facilitate favourable aesthetic outcomes and maintain peri-implant health.^28^ Evidence suggests that restricted accessibility for oral hygiene and an emergence angle of >30 °, combined with a convex emergence profile of the abutment/prosthesis, are associated with an increased risk for peri-implantitis.^29^^,^^30^

### Radiograph protocol

Baseline radiographs at fit of the restoration and following a period of loading are required for reference and to aid with future diagnosis of peri-implant disease.^15^ Some of these radiographs may be unavailable to the GDP. For instance, if the implant was placed and restored in a different practice, it would be advisable (where feasible) to gain a copy of such radiographs through correspondence with the clinician responsible for placing and restoring the implant fixture.

**Fig. 5:**
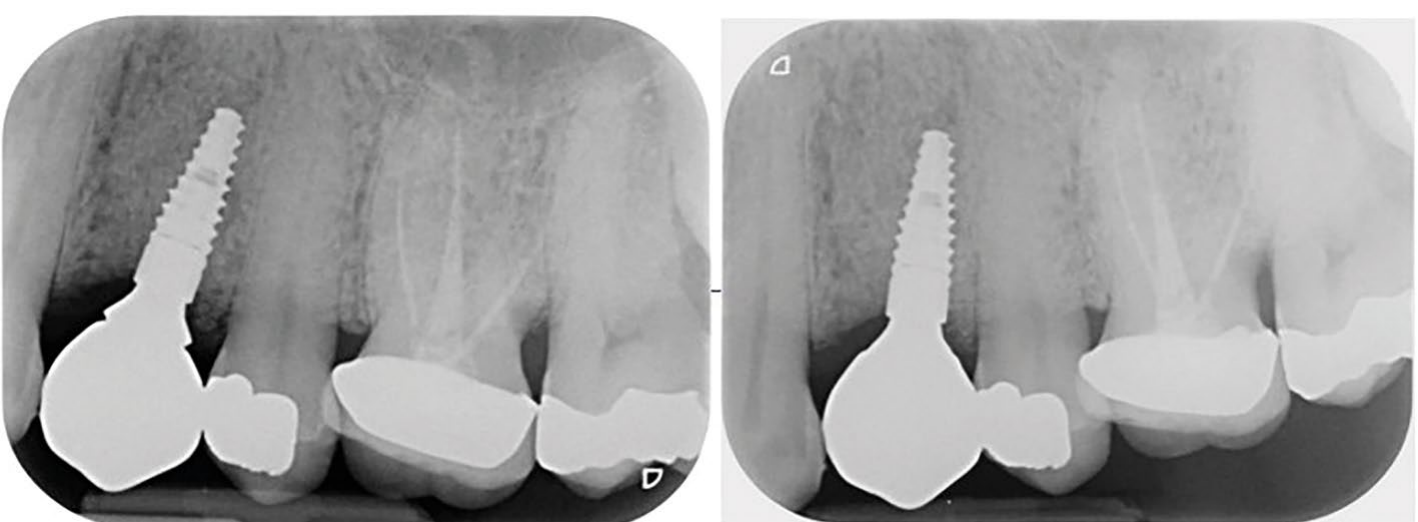
a) PA radiograph taken at a review appointment by the GDP revealing an ill-fitting implant crown resulting in a gap where bacteria can accumulate. b) PA radiograph confirming correct position of the implant crown following adjustment to avoid peri-implant disease

If the GDP is unable to access a copy of baseline radiographs, it would be beneficial to complete a radiographic assessment of the implant when the patient attends for their examination (Fig. 5), as this will provide current status information. In the absence of BOP or increased pocket depths, there is no indication for regular or routine radiographs. Future images need to be justified based on clinical findings. This is because radiographic evidence of bone loss is a late sign of peri-implant disease when compared to the assessment of BOP and pocket depth.^31^ Any sign of marginal bone loss needs to be discussed with the appropriately trained clinician and the patient made aware, as further consideration and investigation may be required to ascertain the cause.

## Diagnosis of peri-implantitis and the role of the GDP

The role of the GDP in peri-implantitis management is in prevention through maintenance of the implant, early detection of peri-implant disease and referral to an appropriately trained clinician for further management. There is now a consensus agreement on the diagnosis of peri-implantitis.^15^ If the baseline radiographs (periapical at fit stage + following a one-year period of loading) and pocket depths are present, then any increase in pocket depth and bone loss in the presence of BOP and/or suppuration can be diagnosed as peri-implantitis.

In the absence of baseline radiographs, the following three clinical findings are indicative of peri-implantitis:^15^

- BOP and/or suppuration, with
- Pocket depth of ≥6 mm, and
- Bone loss ≥3 mm from the neck of the implant.

During the first year after completion of implant treatment, it would be prudent to monitor the patients more frequently (3-4-monthly intervals) in order to ensure early detection of complications. If inflammation is revealed, then any intervention provided needs to continue to be reviewed regularly until stable resolution is achieved, or a referral made to the colleague who placed and restored the implant or an appropriately trained clinician.

If the implant is stable and free of inflammation and complications, after the first year, the reviews can revert to the patient's usual review intervals based on their risk profile for peri-implant disease.

## Conclusion

The key role for the non-implant placing GDP in monitoring implant health is the prevention and early detection of potential peri-implant complications. This is implemented through regular monitoring and maintenance of oral health. Any warning signs detected by using this implant examination checklist should be communicated to the clinician who placed and restored the implant so further investigations or interventions can be implemented sooner. If this is not possible, then a referral to an appropriately trained practitioner would be advised.
